# Transcriptomic Analysis of the Candidate Genes Related to Aroma Formation in *Osmanthus fragrans*

**DOI:** 10.3390/molecules23071604

**Published:** 2018-07-02

**Authors:** Xiu-Lian Yang, Hai-Yan Li, Yuan-Zheng Yue, Wen-Jie Ding, Chen Xu, Ting-Ting Shi, Gong-Wei Chen, Liang-Gui Wang

**Affiliations:** 1 College of Landscape Architecture, Nanjing Forestry University, Nanjing 210037, China; yangxl339@sina.com (X.-L.Y.); tarashiyumi@163.com (H.-Y.L.); yueyuanzheng@njfu.edu.cn (Y.-Z.Y.); ruguoaiduideren@163.com (W.-J.D.); xc127@foxmail.com (C.X.); shitingting313@163.com (T.-T.S.); 18694976599@163.com (G.-W.C.); 2 College of Forestry, Nanjing Forestry University, Nanjing 210037, China

**Keywords:** *Osmanthusfragrans*, transcriptome, floral scent, transcription factor

## Abstract

*Osmanthus fragrans*, or “RiXiangGui”, is an ornamental, woody, evergreen plant that is cultivated widely because it blooms recurrently and emits a strong fragrance. Recently, the germplasm resources, classification, and aroma compositions of *O*. *fragrans* have been investigated. However, the molecular mechanisms of the floral scent formation and regulation have remained largely unknown. To obtain a global perspective on the molecular mechanism of the aroma formation during blooming, nine RNA Sequencing (RNA-Seq) libraries were constructed from three flowering stages: The initial, full, and final flowering stage. In short, a total of 523,961,310 high-quality clean reads were assembled into 136,611unigenes, with an average sequence length of 792 bp. About 47.43% of the unigenes (64,795) could be annotated in the NCBI non-redundant protein database. A number of candidate genes were identified in the terpenoid metabolic pathways and 1327 transcription factors (TFs), which showed differential expression patterns among the floral scent formation stages, were also identified, especially *OfMYB1*, *OfMYB6*, *OfWRKY1*, and *OfWRKY3*, which could play critical roles in the floral scent formation. These results indicated that the floral scent formation of *O*. *fragrans* was a very complex process which involved a large number of TFs. This study provides reliable resources for further studies of the *O.fragrans* floral scent formation.

## 1. Introduction

Sweet osmanthus, a species of the Oleaceae family, is known for its fragrant flowers, which are rich in aromatic flavor when blooming. As one of the top ten traditional Chinese flowers, it has been cultivated for over 2500 years. Through a long evolution, *O*. *fragrans* cultivars were classified into the Thunbergii, Latifolius, Aurantiaeus, and Semperfloren groups. The flowers of the first three cultivar groups bloom in autumn, while the Semperfloren group bloom almost everymonth [1]. “RiXiangGui”, which is a kind of cultivar in the Semperfloren group, which has a high economic value, was cultivated widely due to its important ornamental traits of its floral scent and recurrent blooming.

The floral scent is one of the most important traits of ornamental plants and more than 1700 floral constituents have been identified from 991 species of flowering plants and a few gymnosperms [2]. The components of floral scent are terpenoids, benzenes/phenylpropanes, and fatty acid derivatives [3]. With the rapid development of science and technology, the separation method and the analysis and identification technology of floral constitutes have been promoted using techniques, such as headspace-solid phase, gas chromatography-olfactory, and gas chromatography-mass. According to previous research, the main volatile constituents of sweet osmanthus are terpenoids [4]. The amount of concrete production of *O. fragrans* increases, along with the flower opening, and is at the highest level at the full flowering stage and then decreases at the final flowering stage [4,5,6].

Terpenoids, the largest class of aromatic compounds, are derived from isopentenyl diphosphate and its homologous isomer, dimethylallyl pyrophosphate. They have been synthesized by different terpene synthases (TPSs) via the mevalonate (MVA), or methyl erythritol-4-phosphate (MEP) pathway [7]. The MEP pathway in plant cell plastids has been a key approach for the synthesis of monoterpenes and their derivatives [8]. Xu et al. [9] isolated and identified ten MEP pathway genes in sweet osmanthus. The release regularities of aroma were consistent with the circadian rhythms of the OfDXS2 and OfDHR1 expression patterns. The floral scent released from the flowers or leaves was closely related to the expression level of the TPSs [10,11,12]. The major floral volatiles, linalool, and its derivatives produced in *O. fragrans* flowers, were encoded by OfTPS1, OfTPS2, and OfTPS3 [13]. Moreover, the dioxygenase cleavage step itself could generate volatile products. For example, carotenes were cleaved into α-ionone and β-ionone by OfCCD1 in vitro assays [14]. Except for these, there is no further information available concerning the molecular mechanism of floral scent formation in *O. fragrans*.

Transcription factors (TFs) are proteins combined with *cis*-elements to regulate gene expression both spatially and temporally. They are also involved in the whole process of plant growth and development. Increasing reports indicate that terpene synthesis is governed by transcriptional regulatory networks. In cotton, *GaWRKY1*, bound to the promoter of sesquiterpene cyclase, positively regulates gossypol biosynthesis [15]. *WRKY3* and *WRKY6* have been found to be related to volatile terpene production for defense against pests in tobacco [16]. *MYC2*, a basic helix-loop-helix TF, interacts with the DELLA protein to activate expressions of sesquiterpene synthase genes, *TPS21* and *TPS11*, in *Arabidopsis* inflorescence [17]. *MYB14* participates in the regulation of volatile terpenoid biosynthesis preferentially via the MVA pathway in a conifer tree species [18]. The bZIP (Basic Region-leucine zipper) transcription factor, HY5, interacts with the promoter of the monoterpene synthase gene, QH6, in modulating its rhythmic expression [19]. *Arabidopsis* PAP1, an MYB (V-myb myeloblastosis viral oncogene homolog) TF, can increase the phenylpropanoid-derived color and terpenoids in PAP1-transgenic roses, but the regulatory mechanism remains unclear [20]. *Solanum lycopersicum WRKY73* activates the expression of three monoterpene synthase genes, suggesting that a single WRKY gene could regulate multiple distinct biosynthetic pathways [21]. The discoveries of these TFs provide an attractive strategy for modifying floral substances.

In the model plants, Arabidopsis, Petunia, and other herb plants, such as Snapdragon, Lavender, and Nicotiana, there have been many studies on the molecular mechanisms of the formation of aromatic compounds. However, *O. fragrans* is a woody aromatic plant whose aromatic constitutes are terpenoids. The available gene resources are limited to RNA sequencing (RNA-Seq) technology, an effective tool in genomic exploration and gene discovery in plants when no reference genome is available [22]. It is also capable of providing new insights into the biosynthesis and regulatory mechanisms of floral scents in *O. fragrans*. In this study, nine cDNA libraries of three different flowering stages, including three biological replicates, were constructed in *O. fragrans* for the Illumina RNA-Seq. The analysis of differentially expressed genes (DEGs) and quantitative Real-Time PCR (qRT-PCR) were performed, aiming to excavate interesting genes and TFs associated with aromatic metabolism.

## 2. Results

### 2.1. Sequencing and De Novo Assembly

**Figure 1:**
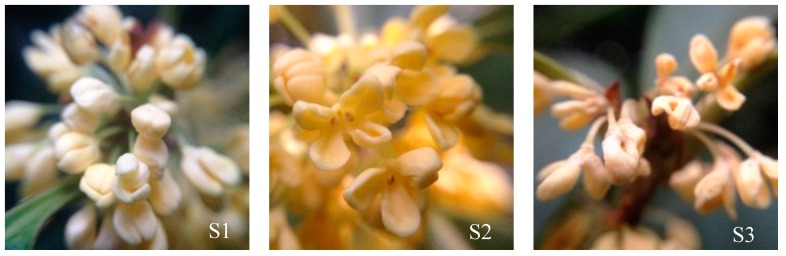
Photographs of flowers at different flowering stages: (**S1**): Initial flowering stage, (**S2**): Full flowering stage, and (**S3**): Final flowering stage.

The flowering process can be divided into four periods, that is, the xiangyan stage, the initial flowering stage, the full flowering stage, and the final flowering stage [23]. The amount of volatiles produced by *O. fragrans* petals gradually increases, along with flower opening and reaches the highest level at the full flowering stage, then declines at the final flowering stage [6]. To obtain genomics resources, to narrow down the special pathways, and to get candidate genes of interest in *O. fragrans*, the petals from three individual plants were collected at the initial flowering (S1), full flowering (S2), and final flowering (S3) stages (Figure 1). In total, nine RNA-Seq libraries were constructed using the above RNA samples and sequenced by Illumina HiSeq™ 4000.

Approximately 532,121,476 raw reads were obtained and 523,961,310 high-quality clean reads remained after filtering. The sequencing result was assembled into a transcriptome using Trinity to act as a reference sequence for the subsequent analysis (Table S1). All transcriptomic data was deposited in the NCBI Sequence Reads Archive (SRA) under the accession number SRP143423. The longest transcript of each gene was a unigene. In total, there were 136,611 unigenes obtained with a maximum length of 16,876 bp, a minimum length of 201 bp, an average length of 792 bp, and an N50 length of 1424 bp (Table 1). The distribution of the unigene lengths is shown in Figure S1.

### 2.2. Annotation and Functional Classification

To determine the putative functions of unigenes, the transcriptome was annotated using protein functions, pathways, KOG (euKaryotic Ortholog Groups) functions, and GO (Gene Ontology) annotations. The unigenes were aligned using the BLASTx program with an E-value threshold of 10^−5^ to the Nr (NCBI non-redundant protein), the Swiss-Prot protein, and the KEGG (Kyoto Encyclopedia of Genes and Genomes) and KOG databases for which the percentages of the annotated unigenes were 42.86, 34.62, 28.58, and 17.44%, respectively (Table 2). Approximately 47.43% of the unigenes were aligned. According to the best alignment results to the species of homologous sequences, the number of homologous sequences of the top ten species is shown in Figure S2.

**Figure 2:**
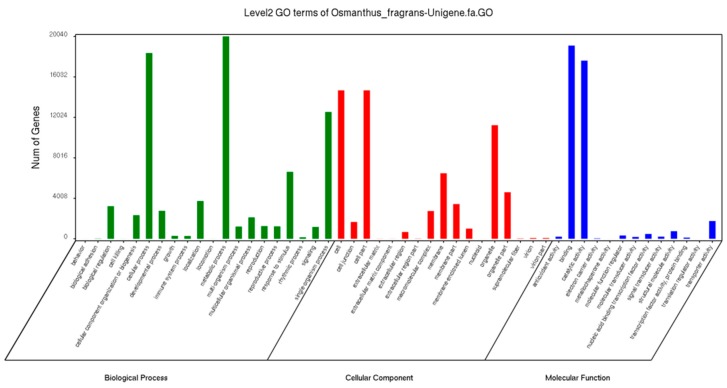
The GO functional classification of differentially expressed genes (DEGs) among different samples.

The GO offered a strictly defined concept to comprehensively describe the properties of genes and their products in any organism. The GO functional annotations of the “RiXiangGui” unigenes were classified into three categories (Molecular Function, Cellular Component, and Biological Process) and 50 subcategories (Figure 2). There were 77,416 (56.67%) unigenes assigned to 20 subcategories in the Biological Process for which “metabolic process”, “cellular process”, and “single-organism process” were the dominant subcategories. The molecular function categories with the most abundant members were “catalytic activity” and “binding”; in the Cellular category, “cell”, “cell part”, and “organelle” were the abundant classes.

In total, 136,611 out of the 39,043 unigenes showing Nr hits were assigned to COG classifications and grouped into 25 categories (Figure S3). The cluster of “general function prediction” was the largest group, representing 35.72%, followed by “post-translational modification, protein turnover, chaperones” and “signal transduction mechanisms”. There were 1537 unigenes in the “secondary metabolites biosynthesis, transport, and catabolism” group.

A total of 23,825 unigenes were annotated and 23,166 sequences were assigned to 132 KEGG pathways (Figure S4). Metabolism (~61.21%) had the largest number of sequences in the primary pathway hierarchy and the unigenes of Genetic Information Processing followed (~27.15%). The flower scents and colors were the main ornamental traits of *O. fragrans*, so we focused on the secondary metabolic pathways, especially those of phenylpropanoids, carotenoids, flavonoids, flavones and flavonol, anthocyanins, and terpenoids. These data provided a valuable resource and narrowed down the special pathways and candidate genes of interest in *O. fragrans*.

### 2.3. Analysis of DEGs

**Figure 3:**
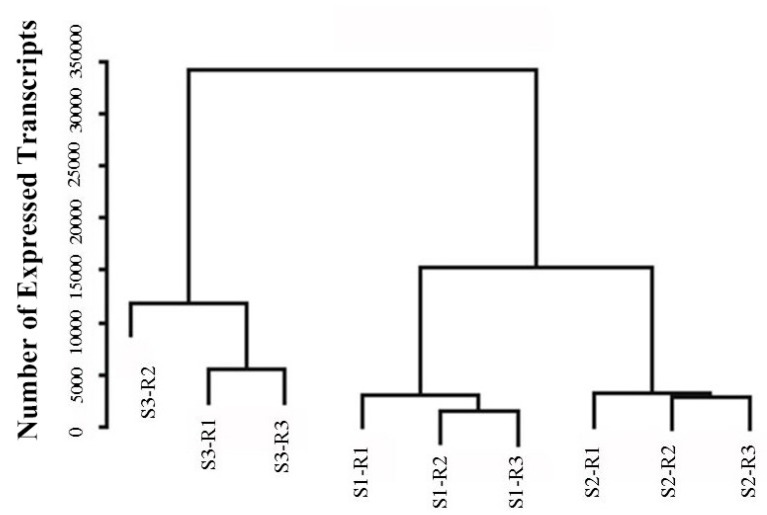
Global analysis of the transcriptome datasets of biological replicates and samples is shown. The dendrogram depicts the global relationships of the samples. The bar plot describes the number of expressed transcripts after filtering.

During the flowering process, the petals of *O. fragrans* release many aromatic compounds. To characterize gene expressions at the S1, S2, and S3 flowering stages, a global expression analysis was performed for each stage using three biological replicates. The DEG libraries were constructed and the unigenes were then mapped to the *O*. *fragrans* reference transcriptome after sequencing by the Illumina platform. The expression of each gene was calculated in RPKM (Reads per kilobase of exon model per million mapped reads). We stratified the relationship of the samples based on the expression level of the whole gene. A dendrogram was plotted and all biological replicates were clustered together (Figure 3). In addition, the results of two parallel experiments to evaluate the reliability and operational stability of the experiments (Figure S5) showed that the correlation index between the two experiments for the same sample was close to 1, indicating a high reproducibility. These revealed that all biological replicas were well correlated and this was similar to S2 and S3.

**Figure 4:**
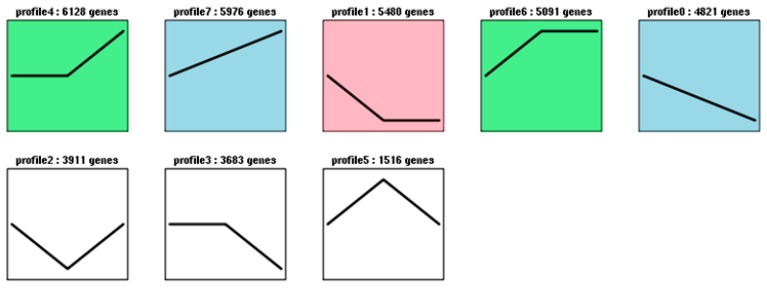
Cluster analysis of the differentially expressed genes. The profiles in color indicate statistical significance (*p* < 0.01). The number on the topis the profile number and assigned genes. The black lines represent the model expression profiles of different genes.

For a better understanding of the gene expression profiles, Short Time-series Expression Miner (STEM) software was used to cluster gene expression patterns. Eight gene clusters with distinct expression patterns were identified (Figure 4). The trend blocks with color indicate significant enrichment trends; clusters7 and 8, containing 5976 and 4821 transcripts, respectively, showed opposite patterns (Figure 4). Cluster 7 showed a gradual increase from S1 to S3, while cluster 8 showed a gradual decrease, indicating that some genes had opposite roles during the flowering process. Cluster 4 (6128 genes) remained constant, then gradually increased, but cluster 6 gradually increased first and then stayed constant. The gene expression profiles revealed that the clusters were involved in biological events during the flowering process.

**Figure 5:**
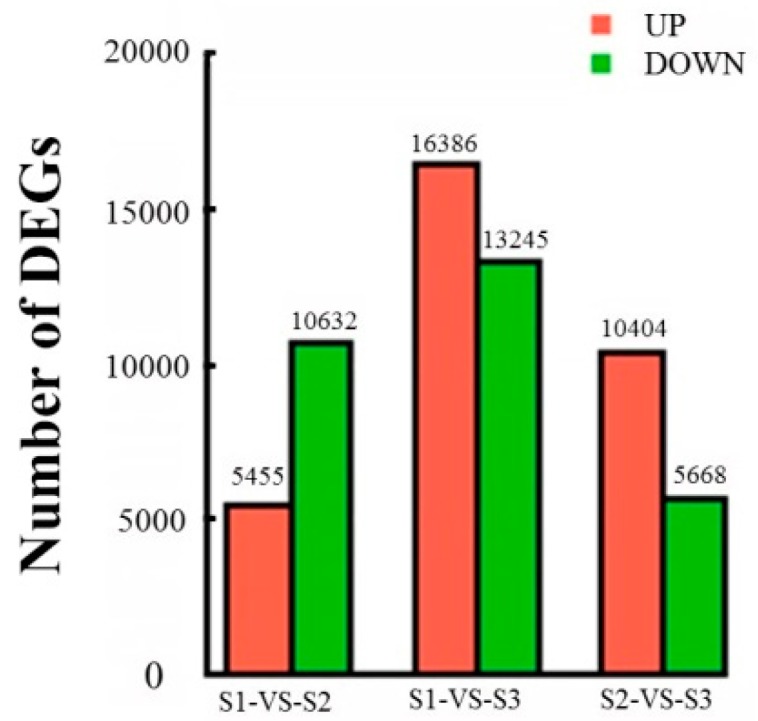
Analysis of differentially expressed unigenes (DEGs) during flowering in *O. fragrans* are listed with different comparisons, including S1versusS2, S1versusS3, and S2versusS3. Red indicates up-regulated unigenes, while green indicates down-regulated unigenes. The *x*-axis indicates the absolute expression levels (Log Conc). The *y*-axis indicates the log-fold changes between the two samples.

To further investigate the DEGs among the groups, transcriptomes generated from different stages were compared. The DEGs were filtered by using a false discovery rate (FDR) < 0.05 and |log_2_FC| > 1. Comparing S1 with S2 resulted in 16,087 DEGs: 5455 were up-regulated and 10,632 were down-regulated. When comparing S1 with S3, there were 29,631 DEGs: 16,386 were up-regulated and 13,245 were down-regulated. The comparison of S2 with S3 showed 16,072 DEGs: 10,404 were up-regulated and 5668 were down-regulated (Figure 5).

### 2.4. Identification of UnigenesInvolved in Scent Metabolism

**Figure 6:**
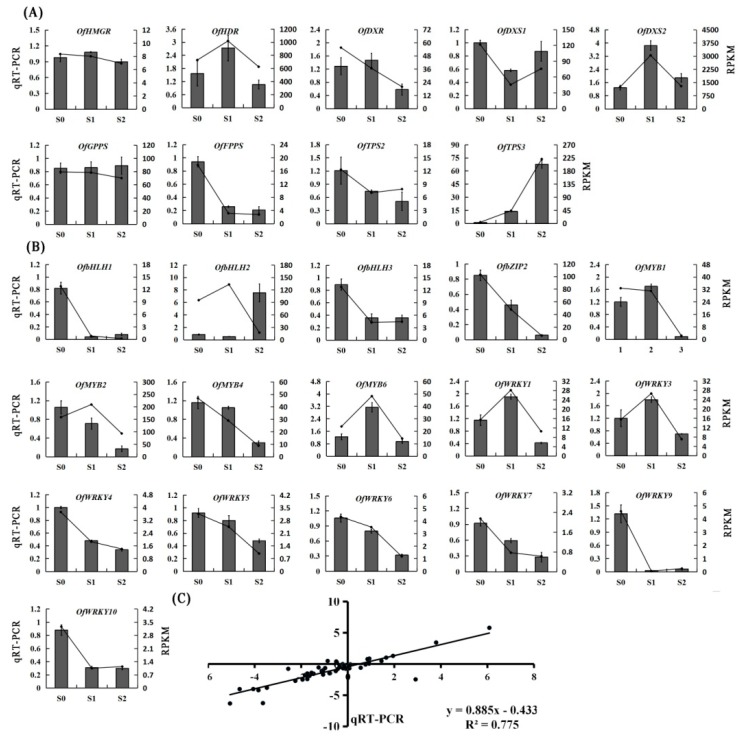
The qRT-PCR validation of DEGs. (**A**) Expression patterns of genes encoding enzymes possibly involved in terpenoid biosynthesis; (**B**) Expression analysis of TFs in different flowering stages. Relative transcription level of flowers was set to be 1 (100%). Error bars indicate the calculated maximum and minimum expression quantity of the three replicates. Different lowercase letters labeled on the bars indicate statistically significant differences at the level of *p* < 0.05. S1, initial flowering stage; S2, full flowering stage; S3, final flowering stage; (**C**) The correlation analysis of the gene expression ratios between qRT-PCR and RNA-seq.

In previous studies, the *O. fragrans* floral constituents have been found to be volatile terpenoids. The terpenoid metabolic pathway genes, and many of the biosynthetic enzymes involved, have been identified and functionally characterized. The key genes involved in the terpenoid pathway were identified according to the KEGG annotation and a local BLASTx search (Table 3). A total of nineunigenes, involved in terpenoid biosynthesis, were selected to validate the sequencing data. The qRT-PCR was carried out with independent samples collected from the different flowering stages to analyze their differential expression pattern (Figure 6A). The primer sequences used arelisted in Table S2.

### 2.5. Analysis of TFs during Blooming

TFs are crucial in regulating the gene expressions involved in plant growth, development, and other physiological functions. According to the searches against the Pln TFDB (Plant Transcription Factor Database), 2269 TF genes were obtained and classified into 57 TF families. The top ten families were C2H2 (234), ERF (193), bHLH (168), MYB-related (155), NAC (134), WRKY (134), MYB (96), bZIP (89), GRAS (82), and C3H (73). The number of the TFs identified in differential expression profiles is shown in Table 4. The MYB and WRKY families have been shown to be involved in regulating secondary metabolisms in plants, especially floral volatiles and pigments. Significant differential expression TFs were selected to perform the qRT-PCR. A few of the TF family members had the highest expression levels at the initial flowering stage and the lowest expression levels at the final flowering stage (Figure 6B). Interestingly, the expression level of *OfMYB1*, *OfMYB6*, *OfWRKY1*, and *OfWRKY3* reached the highest at the full flowering stage and declined at the final flowering stage, which was consistent with the release of the floral volatiles (Figure 6B).

### 2.6. Validation of RNA-Seq Results by qRT-PCR

Nine key genes were selected in the terpenoid metabolic pathway and 16 TFs for the qRT-PCR to verify the sequencing data. The primers of the TFs were designed using the Primer 5.0 software and areshown in Table S3. The expression levels of these selected TFs in different flowering stages isdepicted in Figure 6B. The results showed that the expression patterns of these candidate genes obtained by qRT-PCR were largely consistent with RNA-Seq. The linear regression analysis revealed that the fold change values of qRT-PCR and RNA-Seq showed a strong positive correlation (R^2^ = 0.775) at the level of *p* ≤ 0.01 (Figure 6C). These results indicate that the RNA-Seq data were reliable.

## 3. Discussion

*Osmanthus fragrans*, “RiXiangGui”, a traditional horticultural plant known for its floral scent, is widely planted in Asia, especially in southern and central China [24]. Owing to its appealing fragrance and, potentially, exploitable chemical substances, the flowers areextensively used to produce teas, foods, perfumes, and medicines [6]. However, little information is available to explain the transcriptional regulation of the floral scent genes. As far as we know, there have been only two previous transcriptome reports based on RNA-Seq technology on the Oleaceae family. Using an orange-red-flowered cultivar, “YanhongGui”of the *O. fragrans*, the petal color, carotenoid content, transcriptome dynamics of flower buds, and expression of key genes for the carotenoids were analyzed [25]. In our previous study, 197 and 237 candidate genes involved in fragrance and pigment biosynthesis, respectively, were functionally annotated [26]. Here, nine libraries were constructed to perform RNA-Seq, and 136,611 unigenes were generated with a maximum length of 16,876 bp, a minimum length of 201 bp, an average length of 792 bp, and an N50 length of 1424 bp. Due to the lack of available genetic information, only 47.43% of the unigenes searched against the Nr database were matched, suggesting that a large portion of the genes acting during blooming might involve some unique processes and pathways. The annotated unigenes of *O*. *fragrans* showed the highest homology tothe unigenes of *Sesamum indicum* (Figure S2), which may reflect a close evolutionary relationship between the two species.

Plants release an array of secondary metabolites to adapt to changing environments throughout their life cycles. Floral volatile organic compounds are a relatively large group, which plays crucial roles in pollinator attraction, defense, communication, and interaction with the surrounding environment. They are classified into three major groups: Terpenoids, phenylpropanoids/benzenoids, fatty acid derivatives [27]. Terpenoids are the largest class of floral volatiles and encompass 556 scent compounds [28]. Flower scents vary between plant species because their different relative proportions of volatile compounds. Within a species, the blend of emitted terpenoids differs quantitatively and qualitatively, with some compounds having common properties [29]. The *β*-ionone, *cis*-linalool oxide (furan), *trans*-linalooloxide (furan), and linalool were abundant in most *Osmanthus fragrans* cultivars, with other compounds typically present in smaller amounts [4].

The biosynthetic enzymes involved and the emission of terpenes have been identified and functionally characterized in many plants, such as Arabidopsis [30], Snapdragon [31,32], *Clarkia breweri* [33,34,35,36], Petunia [37], and Rose [38,39]. Terpenoids, derived from prenyl diphosphate precursors, are synthesized by two independent pathways: The MVA pathway in the cytoplasm and the MEP pathway in plastids [40]. Based on sequence annotations and the analysis of changes in gene expressions during each flowering stage, key floral scent-synthesizing genes, *DXS*, *DXR*, *HDR*, *TPS*, and *GPPS*, were identified. The results indicated that the expressions of *DXS*, *DXR*, *HDR*, and monoterpene synthases were all positively correlated with monoterpene emission. *DXS* was the first gene identified and is thought to be an important rate-controlling step of the MEP pathway [8]. Two *OfDXSs* showed differential expression patterns that the transcript level of *OfDXS1* was down-regulated at the full flowering stage, while *OfDXS2* increased sharply. It seemed that the *OfDXS2* transcript was correlated with monoterpene emission. These genes showed similar expression patterns in *Hedychiumcoronarium* [41]. *DXR* may also serve as a significant control factor of the metabolic flux through the MEP pathway, since it catalyzed the first committed step of the pathway towards terpenoid biosynthesis [42,43]. *HDR* catalyzed the last step of the MEP pathway and the highest expression was at the full flowering stage. The TPSs were directly responsible for the synthesis of various terpenes, *OfTPS2* and *OfTPS3,* andhad almost opposite expression patterns at each flowering stage.

A total of 1327 TF genes were differentially expressed in the *O. fragrans* transcriptome. The role of transcriptional regulation in reprogramming metabolic processes during plant development and in response to biotic and abiotic cues has been recognized in several plant systems. Among the regulatory networks governing secondary metabolisms, there have been many studies on flavonoid biosynthesis, but very fewwere known for the regulation of floral volatile production at the molecular level [44,45,46,47]. Only a few MYB and WRKY TF family membershave been characterized and shown to have crucial roles in regulating the biosynthesis of floral volatiles. To date, of the R2R3-MYB TF family, *PhODO1*, *PhEOBI*, *PhEOBII*, and *PhMYB4* have been characterized as regulators of volatile phenylpropanoids in petunia [48,49,50,51]. The *AtPAP1* transcription factor enhanced the production of phenylpropanoid and terpenoid scent compounds in rose flowers [20]. MYC2, a bHLH TF, directly bound to the promoters of the sesquiterpene synthase genes, *TPS21* and *TPS11*, and activated their expression in *Arabidopsis* flowers [17]. In the WRKY family, *GaWRKY1*, *NaWRKY3*, *NaWRKY6*, and *SlWRKY73* were found to be related to volatile terpenoid production [15,16,21].

The qRT-PCR analysis revealed that the *bHLH*, *MYB*, and *WRKY* family members were differentially expressed at each flowering stage. The expression patterns of *OfMYB1*, *OfMYB6*, *OfWRKY1*, and *OfWRKY6* were consistent with the release of floral volatiles, which may play critical roles in regulating the formation of floral scent. Further experiments, including yeast one-hybrid and trans-activation assays using promoters of key structural genes, need to be carried out to determine their involvement in floral scent production.

## 4. Materials and Methods

### 4.1. Plant Materials and RNA Extraction

Plants of *O. fragrans*, “RiXiangGui”, cultivated over decades, were located in the Nanjing Forestry University campus, Jiangsu, China. Three plants (R1, R2, R3) grown under the same conditions were randomly selected as biological replicates. The flowers at initial flowering (S1), full flowering (S2), and final flowering (S3) stages were collected and immediately frozen in liquid nitrogen and stored at −80 °C until use (Figure 1).

The RNA of the samples was extracted using a total RNA extraction kit according to the manufacturer’s protocol (Tiangen Biotech, Beijing, China). The RNA quantity was analyzed by NanoDrop 2000 and the quality was determined by 1.2% agarose gel electrophoresis. For each developmental stage, equal amounts of high-quality RNA from the three plant samples were constructed into nine cDNA libraries for Illumina deep sequencing.

### 4.2. Libraries Construction and Illumina Sequencing

After the total RNA was extracted in the three stages of flowering containing three biological replicates for each stage, the eukaryotic mRNA was enriched by Oligo (dT) beads. Then, the enriched mRNA was fragmented into short fragments using a fragmentation buffer and reverse transcripted into cDNA with random primers. The second-strand cDNA was synthesized by DNA polymerase I, RNase H, dNTP, and a buffer. The cDNA fragments were purified with a QiaQuick PCR extraction kit, eluted by EB buffer, end repaired, poly (A) added, and ligated to the Illumina sequencing adapters. The ligation products were size-selected by agarose gel electrophoresis and amplified by PCR. Finally, all the libraries were sequenced using IlluminaHiSeq™4000 (Gene Denovo Biotechnology Corporation. Guangzhou, China).

### 4.3. Assembly and Functional Annotation

To get high-quality clean reads, raw reads were filtered by removing adapters, reads containing more than 10% of unknown nucleotides (N), and low-quality reads containing more than 50% of the low-quality bases (Q-value ≤ 10). At the same time, Q20, Q30, GC-content, and the sequence duplication level of the clean data were calculated. The de novo assembly of the transcriptome was carried out with the Trinity software, which was used for the subsequent analysis without a reference genome [52]. All the assembled unigenes were aligned to public databases, by BLASTX (http://www.ncbi.nlm.nih.gov/BLAST/) with an E-value threshold of 10^−5^, including the NCBI non-redundant protein (Nr) (http://www.ncbi.nlm.nih.gov), Swiss-Protprotein (http://www.expasy.ch/sprot), Kyoto Encyclopedia of Genes and Genomes (KEGG) (http://www.genome.jp/kegg), and COG/KOG (http://www.ncbi.nlm.nih.gov/COG) databases. The classification of the Gene Ontology (GO) was analyzed using the Blast2GO software (http://www.blast2go.com) [53]. 

### 4.4. Gene Expression Analysis

Every sample of the clean reads was mapped back onto the assembled transcriptome by the bowtie [54]. The differential expression analyses of each of the two groups were performed using the DESeq R package. The results of the aligned clean reads were counted and quantified to give the expression abundance of gene expression using the RSEM package (Reads Per Kilo bases per Million mapped Reads) [55]. Correlation analyses of the biological replicates were performed. Differences in the expression analysis between the two samples were determined using the DESeq R package. The threshold to select the DEGs was set as follows: qvalue < 0.005 and |log_2_ (foldchange)| > 1. Clusters of differentially expressed genes were analyzed using the STEM software (http://www.cs.cmu.edu/jernst/stem/) [56] and the profiles in color indicated statistical significance (*p* < 0.01). The GO enrichment analysis of the DEGs was implemented by the GOseq R packages based on the Wallenius non-central hypergeometric distribution [57], which could adjust for gene length bias in the DEGs. The KOBASs of software was used to test the statistical enrichment of DEGs in the KEGG pathways [58].

### 4.5. qRT-PCR Validation

Nine DEGs involved in terpenoids were chosen for qRT-PCR validation. The first-strand cDNA was reverse transcribed with 5 µg of total RNA isolated from each of the three flowering stages and biological replicates using a RevertAid First Strand cDNA Synthesis Kit (Thermo Fisher Scientific, Wilminton, DE, USA). The primers, designed using the Primer Premier 5.0 software (Premier Biosoft International, Palo Alto, CA, USA), are listed in Table S2. TheqRT-PCR was performed on 10 µL reactions systems containing a cDNA template, forward and reverse primers, SYBR Premix Ex Taq (TaKaRa, Ostu, Japan), and water, with an ABI Step One Plus Systems (Applied Biosystems, Carlsbad, CA, USA). The PCR reaction conditions were as follows: 95 °C for 30 s, followed by 40 cycles of 95 °C for 5 s, 60 °C for 30 s, and 95 °C for 15 s. *OfRPB2* was used as the reference gene to calculate the relative fold-differences based on the comparative cycle threshold (2^−ΔΔCt^) values [59]. Every reaction for each gene used three biological replicates, with three technical replicates.

## 5. Conclusions

The dynamic transcriptome sequencing analysis of “RiXiangGui” at different flowering stages was performed using Illumina RNA-Seq technology. Based on the DEG analysis and previous research onthe patterns of the release of floral compounds, several candidate genes related to monoterpenes, sesquiterpenes, and phenylpropanoid biosynthesis were obtained. Furthermore, a series of TFs were identified and theqRT-PCR analysis indicated that *OfMYB1*, *OfMYB6*, *OfWRKY1*, and *OfWRKY6* participate in terpenoid biosynthesis. These results are helpful for exploring the molecular mechanism of floral scent formation in *O. fragrans*, and supplies important gene resources for the floral scent breeding of ornamental plants.

## Figures and Tables

**Table 1:** The Summary of the *O. fragrans* transcriptome.

Transcript	Unigene
Total assembled bases	108,311,010
Total number of genes	136,611
Max length of unigenes (bp)	16,876
Min length of unigenes (bp)	201
Average length ofunigenes (bp)	792
N50 (bp)	1424
GC percentage (%)	38.9215

**Table 2:** The statistics of annotation on unigenes against public databases.

Database	Number of Annotated Unigenes	Percentage of Annotated Unigenes (%)
Nr	58,556	42.86
Swiss-prot	47,294	34.62
KOG	39,043	28.58
KO (KEGG Orthology)	23,825	17.44
Total	64,795	47.43

**Table 3:** The KEGG enrichment of DEGs among the three flowering stages.

KEGG Pathway	All Genes with Pathway Annotation	DGEs Genes with Pathway Annotation	Pathway ID
Phenylpropanoid	319	178	Ko00940
Monoterpenoid biosynthesis	17	8	Ko00902
Terpenoid backbone biosynthesis	184	78	Ko00900
Sesquiterpenoidand triterpenoid biosynthesis	69	45	Ko00909
Diterpenoid biosynthesis	45	25	Ko00904
Limonene and pinene degradation	15	10	Ko00903

**Table 4:** The number of the transcription factors identified in the expression profiles.

Transcription Factors	Profile0	Profile1	Profile4	Profile6	Profile7	All Profiles
C2H2	3	2	1	0	2	234
ERF	56	88	50	21	50	193
bHLH	24	26	13	5	4	168
MYB-related	4	6	6	3	5	155
NAC	101	158	110	47	80	134
WRKY	3	4	41	7	24	112
MYB	18	13	24	9	19	96
bZIP	4	3	2	0	0	89
GRAS	2	0	8	11	15	82
C3H	0	1	0	0	2	73
FAR1	3	6	11	6	10	67
Dof	11	3	2	0	1	59
G2-Like	3	0	1	1	1	56
HD-ZIP	0	1	0	0	0	52
Trihelix	4	2	2	0	3	50
HSF	2	1	0	1	0	43
TCP	6	11	2	0	7	41
B3	6	11	10	8	8	40
GATA	14	10	0	1	3	34
SBP	4	5	0	0	0	32
ARF	0	2	2	1	1	29
M-type	4	3	11	5	7	22
NF-YC	0	1	0	0	0	16
AP2	4	7	5	2	1	14
CPP	0	1	0	0	0	12
WOX	1	1	0	0	0	11
LSD	0	0	1	0	0	9
YABBY	1	1	0	0	0	8
BES1	2	2	0	0	0	8
SRS	1	0	1	2	1	6

## Supplementary Materials

The following are available online. Table S1. The summary of transcriptome sequencing data and transcriptome assembly; Figure S1. The length distribution of assembled *O. fragrans* unigenes. All the Illumina reads for each flowering stage were combined together with 136,611 transcripts obtained. The horizontal and vertical axes showed the size and the number of transcripts, respectively; Figure S2. The characteristics of homology search of unigenes against the Nr database; Figure S3. The Eu Karyotic Orthologous Groups (KOG) classifications in *O. fragrans*. A total of 136,611sequences with KOG classifications within the 25 categories were shown; Figure S4. The summary of the KEGG pathways involved in the *O. fragrans* flower transcriptome. A: Metabolism; B: Genetic Information Process; C: Environment Systems; D: Cellular Process; E: Organismal Systems; Figure S5. A heat map plot of nine clusters displaying the relative expression levels of centroids; Table S2. The primers used for qRT-PCR; Table S3. The primers of TFs used for qRT-PCR.
