# Supplementary material for: Transcriptomic Analysis of the Candidate Genes Related to Aroma Formation in Osmanthus fragrans
Source: Molecules. 2018 Jul 2;23(7):1604. doi: 10.3390/molecules23071604 (PMC6100529; doi:10.3390/molecules23071604)
Supplement: Supplementary file 1 [file molecules-23-01604-s001.pdf]

molecules

MDPI

Table S1. The summary of transcriptome sequencing data and transcriptomeassembly.

Sample	Raw Reads	Clean Reads	GC (%)	Adaptor (%)	Low Quality (%)	Q20 (%)	Q30 (%)
S1-R1	56,727,396	55,846,862	43.94%	0.04%	1.15%	97.93	94.76
S1-R2	61,833,244	60,966,256	44.11%	0.04%	1.01%	98.03	94.96
S1-R3	86,376,464	84,598,042	44.05%	0.03%	1.67%	97.62	94.04
S2-R1	46,858,546	46,188,794	43.86%	0.03%	1.03%	98.02	94.93
S2-R2	55,732,750	54,949,688	43.36%	0.03%	1.01%	98.06	95.02
S2-R3	54,468,388	53,697,708	43.37%	0.03%	1.02%	98.03	94.94
S3-R1	57,508,808	56,711,496	43.52%	0.04%	0.99%	98.04	94.98
S3-R2	56,463,786	55,663,954	43.61%	0.03%	1.02%	97.96	94.79
S3-R3	56,152,094	55,338,510	43.24%	0.03%	1.06%	97.98	94.83
Total	532,121,476	523,961,310					

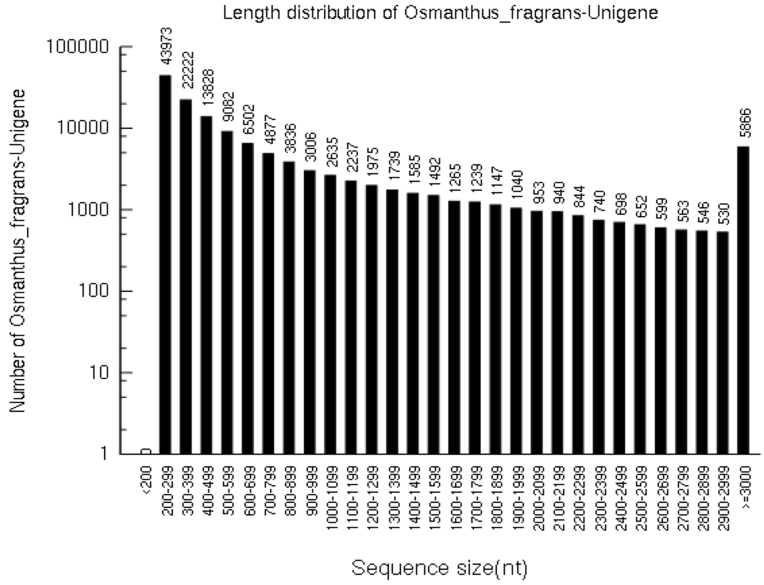

Figure S1. The length distribution of assembled *O. fragrans* unigenes. All the Illumina reads for each flowering stage were combined togetherwith136,611 transcripts obtained. The horizontal and vertical axes showed the size and the number of transcripts, respectively.

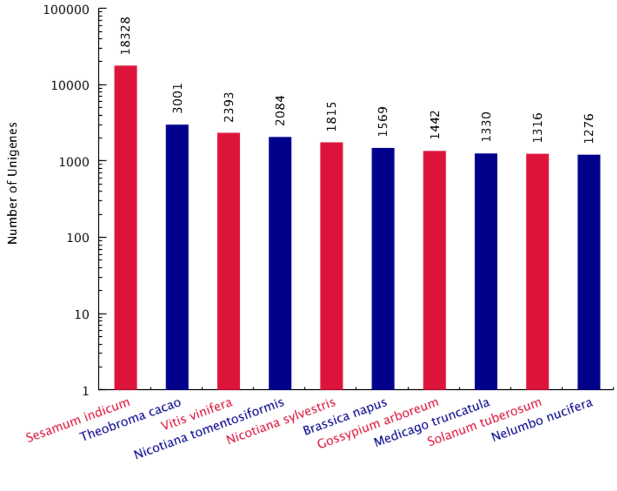

Figure S2. The characteristics of homology search of unigenes against the Nr database.

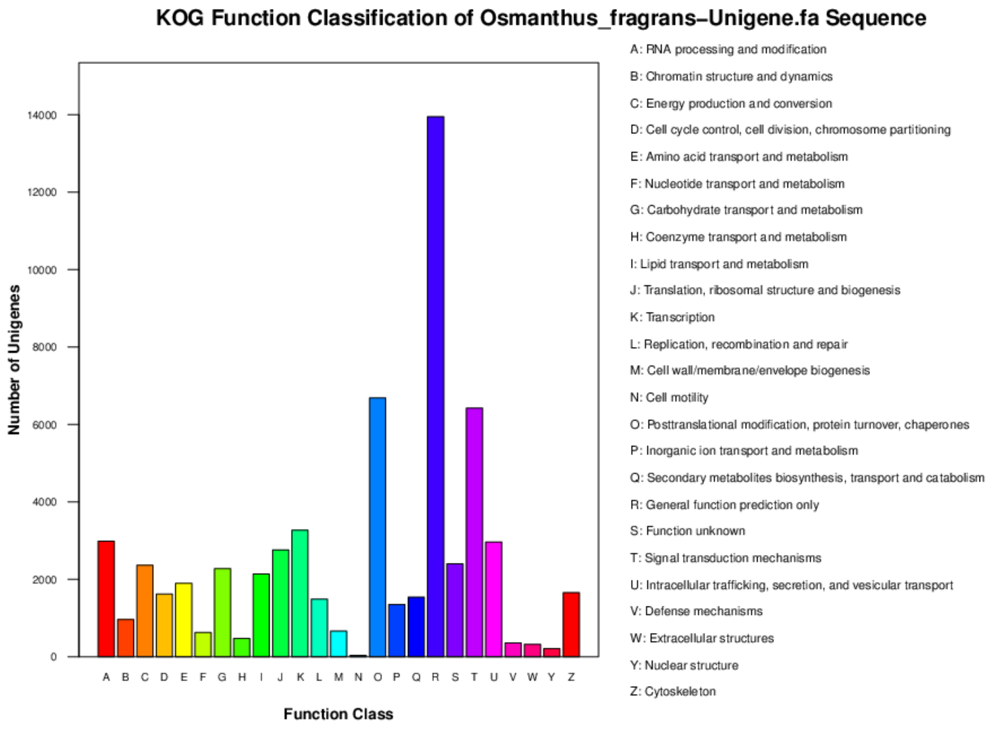

Figure S3. The Eu Karyotic Orthologous Groups (KOG) classifications in *O. fragrans*. A total of 136,611sequences with KOG classifications within the 25categories were shown.

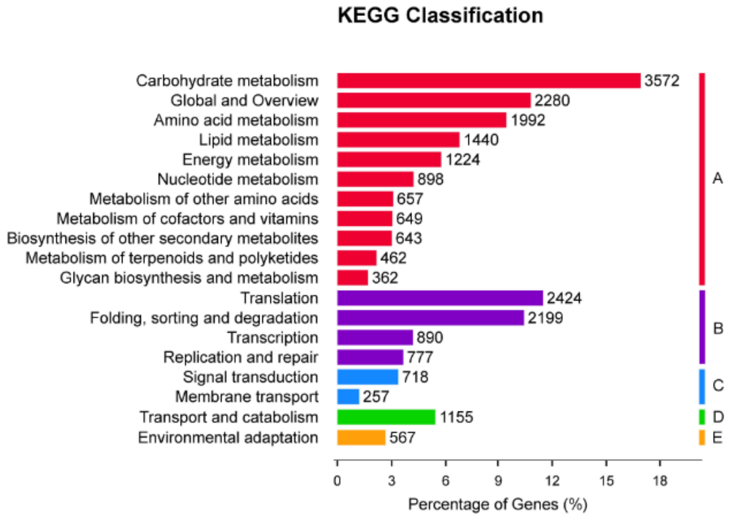

Figure S4. The summary of the KEGG pathways involved in the *O. fragrans* flower transcriptome. A: Metabolism; B: Genetic Information Process; C: Environment Systems; D: Cellular Process; E: Organismal Systems.

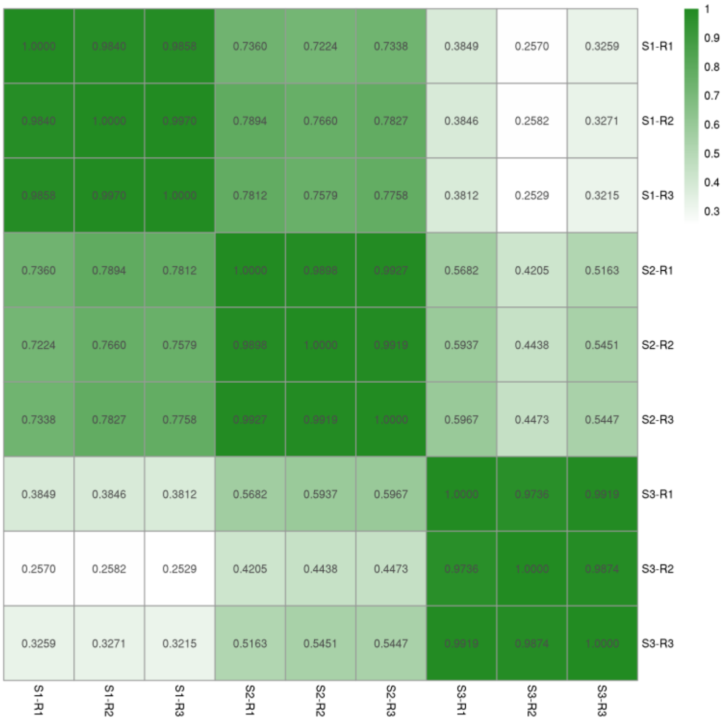

Figure S5. A heat map plot of nine clusters displaying the relative expression levels of centroids.

Table S2. The primers used for qRT-PCR.

Gene	Description	Forward Primer (5′ to 3′)	Reverse Primer (5′ to 3′)
OfTPS2	Terpene synthase	GACCATCTCCTGTTGCGTGTAG	TCAATAATTCCCTAACATCCTTC AGT
OfTPS3	Terpene synthase	ATGGCACTTACCACCCTACTCC	TTCCCCACCTTCGTTCGG
OfDXS1	1-Deoxy-D-xylulose5-phosphate synthase	AGTCACCGAGAAAGGCAGA	GGAAGCGACGCAAGAAAA
OfDXS2	1-Deoxy-D-xylulose5-phosphate synthase	GGGCATTTGATGTAGCATA	ATCCTTCCCTTACCAATCT
OfDXR	1-Deoxy-D-xylulose 5-phosphate reductoisomerase	CACCTTCTTCTCCCTCGTCCT	CAACTATTACAGCCACCAATCTC C
OfHDR2	4-hydroxy-3-methylbut-2-enyl diphosphate reductase	ACAATCGGAAGGGGTTT	TTCTCGCCTCGTAAGCA
OfGPPS	geranyl pyrophosphate synthase	TCCGAGTTCGTTTCGTTTAGC	TTTCAGGATTACGGATTACCCA
OfHMG R	Hydroxymethylglutaryl-CoA reductase	GCTCCTCCCACGACGCTT	GGATTTCGCCCGAGACCA
OfFPPS	Farnesylpyrophosphate synthase	TGAAGACGCAGGCACATTTATT	TCCAGGTACATTGTAGTCCAACA TC

Table S3. The primers of TFs used for qRT-PCR.

Gene	Forward Primer (5′ to 3′)	Reverse Primer (5′ to 3′)
OfbHLH1	CGAAGTCATAGTGAATCGTGGC	GAAGTGTTCTTTCATTTGCTTTTGTA
OfbHLH2	GCACTTGGGTTCTGTTCTTCAC	TTTGGAGTCGAACGCTAATGG
OfbHLH3	CTTGAGTTGAGGGTATCCCACAT	GCAGTGATGTTGGCAGTAATGAT
OfbZIP2	TTTCCATAAAGGATTTTGAGGG	CTCATTCGGGATCGTTTCG
OfMYB1	CAGGTTACTGAGGCTTTGCG	CTTTGATAGCCAGTTGCGAGA
OfMYB2	CACTGGAATACCCGTATGCTCA	CGCTTGCGTTCAACTCCTG
OfMYB4	GCAGCGGAGGTTGAGGAG	ACTTGACGATGAACCAGAGCC
OfMYB6	CAATCCAGTCTCGGGCACC	CAACCTGGGATGGAATAGCC
OfWRKY1	AAAACTCCCGAGCTGGTAAGA	GGATACTGGCTGCCTTTGACT
OfWRKY3	GGCGGTGGAAATGATGGC	CGCTCTGGTGCTGGAGTTTG
OfWRKY4	GGTCCCTCCTCCTATTGATGC	CACAAGAAACCTGAGAAAACGAG
OfWRKY5	AGCCGATGATGCGGATAAA	TCCTCAAGACCCACAAAGAAAT
OfWRKY6	TGCTCCAACCCCATAACCA	TTCGGTTGTGGATAGAAATGGAGT

OfWRKY7	TCCCCGAGACGAACTTTGA	ACTCCCTCCACCTGTGCTACTA
OfWRKY9	GATTTGCGGTGCCCAGAC	CCCAACCTTGAGACCCCTTAT
OfWRKY10	TGAACAACCGAAACGCCATC	GCCACTATTACTACGCCGACAC

© 2018 by the authors. Licensee MDPI, Basel, Switzerland. This article is an open access article distributed under the terms and conditions of the Creative Commons Attribution (CC BY) license (http://creativecommons.org/licenses/by/4.0/).
